# PLAGL2 promotes epithelial–mesenchymal transition and mediates colorectal cancer metastasis via β-catenin-dependent regulation of ZEB1

**DOI:** 10.1038/s41416-019-0679-z

**Published:** 2019-12-12

**Authors:** Liang Wu, Zili Zhou, Shengbo Han, Jinhuang Chen, Zhengyi Liu, Xudan Zhang, Wenzheng Yuan, Jintong Ji, Xiaogang Shu

**Affiliations:** 1 Department of Gastrointestinal Surgery, Union Hospital, Tongji Medical College, Huazhong University of Science and Technology, Wuhan, 430022 China; 2 Department of Emergency Surgery, Union Hospital, Tongji Medical College, Huazhong University of Science and Technology, Wuhan, 430022 China; 3 Department of Gastrointestinal Surgery II, Renmin Hospital of Wuhan University, Wuhan, 430060 China; 4 Department of Gastrointestinal Surgery, The Central Hospital of Wuhan, Tongji Medical College, Huazhong University of Science and Technology, Wuhan, 430022 China

**Keywords:** Colorectal cancer, Oncogenes

## Abstract

**Background:**

We previously demonstrated that the pleomorphic adenoma gene like-2 (PLAGL2) is involved in the pathogenesis of Hirschsprung disease. Enhanced PLAGL2 expression was observed in several malignant tumours. However, the exact function of PLAGL2 and its underlying mechanism in colorectal cancer (CRC) remain largely unknown.

**Methods:**

Immunohistochemical analysis of PLAGL2 was performed. A series of in vitro and in vivo experiments were conducted to reveal the role of PLAGL2 in the progression of CRC.

**Results:**

Enhanced PLAGL2 expression was significantly associated with EMT-related proteins in CRC. The data revealed that PLAGL2 promotes CRC cell proliferation, migration, invasion and EMT both in vitro and in vivo. Mechanistically, PLAGL2 promoted the expression of ZEB1. PLAGL2 enhanced the expression and nuclear translocation of β-catenin by decreasing its phosphorylation. The depletion of β-catenin neutralised the regulation of ZEB1 that was caused by enhanced PLAGL2 expression. The small-molecule inhibitor PNU-74654, also impaired the enhancement of ZEB1 that resulted from the modified PLAGL2 expression. The depletion of ZEB1 could block the biological function of PLAGL2 in CRC cells.

**Conclusions:**

Collectively, our findings suggest that PLAGL2 mediates EMT to promote colorectal cancer metastasis via β-catenin-dependent regulation of ZEB1.

## Background

Colorectal cancer (CRC) is the third most frequent cancer worldwide, ranking second in cancer-related mortality.^1,2^ Metastasis, accounting for up to 90% of cancer-related deaths, is still the most incomprehensible part of cancer progression.^3^ Evidence is mounting that epithelial–mesenchymal transition (EMT) initiates the metastatic progression of CRC.^4–6^ EMT is a transdifferentiation process, that is associated with enhanced tumour dissemination, disruptions the apical-basal polarity, decreased numbers of cellular junctions, and EMT requires the reduction of E-cadherin expression. During EMT, differentiated epithelial cancer cells from the primary tumour, lose their epithelial characteristics and assume a mesenchymal phenotype, which promotes the formation of an invasive phenotype and enhances cancer cell metastasis. The molecular characteristics of EMT include the suppression of epithelial markers, including E-cadherin, and the concomitant promotion of mesenchymal markers such as N-cadherin and vimentin.^7^ In the initiation of EMT, E-cadherin depletion is a crucial initial step.^4^ Various EMT-inducing transcription factors, including Snail, Twist and ZEB protein families, and corresponding intracellular signalling pathways can initiate the EMT process.^8^ There are the most consistent negative correlations between the expression levels of ZEB1 and E-cadherin in various cancers.^9^ In EMT activation, ZEB1 not only suppresses epithelial gene expression but also upregulates mesenchymal markers such as N-cadherin. ZEB1 expression is also associated with worse clinical outcomes across different types of tumours.

Signals, such as the TGFβ and Wnt/β-catenin pathways, induce EMT by triggering the expression of Snail1 and ZEB1.^8^ One of the most important signalling pathways in the induction of EMT is the Wnt/β-catenin signalling pathway, which promotes the nuclear translocation of the oncoprotein β-catenin. The β-catenin nuclear accumulation can be observed in approximately 80% of CRC specimens.^10^ β-Catenin that is located in the nucleus functions as a coactivator of T-cell and lymphoid enhancer factors (TCF–LEFs) to transcriptionally activate downstream genes.^9^ The abnormal activation of β-catenin/TCF signalling has been implicated in various tumours, most notably CRC. Due to the inactivated Wnt ligand, cytoplasmic β-catenin is phosphorylated by a complex with GSK-3β, APC and Axin, and is degraded by the proteasome and then prevented from reaching the nucleus.^11^ Due to a lack of nuclear β-catenin, TCF–LEFs instead act as transcriptional repressors.^9^

PLAGL2, containing a C2H2 zinc finger, serves a carcinogenic function and is involved in the pathogenesis of numerous tumours.^12–16^ In addition, the PLAG family proteins (PLAG1, PLAGL1, and PLAGL2), have highly homologous N-terminal zinc finger structures.^13^ PLAGL2 and PLAG1 are oncogenes involved in various malignancies, whereas PLAGL1 functions as a tumour suppressor.^13^ Aberrant PLAG1 expression is involved in the development of uterine leiomyomas^17^ and salivary gland tumours.^18^ The overexpression of PLAGL2 contributes to the development of malignant gliomas by strongly impeding their differentiation and by promoting their self-renewal capacity.^12^ Growing evidence has demonstrated that enhanced PLAGL2 expression functions as a dominant oncogene in gastrointestinal cancers.^19^ In CRC, PLAGL2 is one of the top 20 overexpressed genes at 20q11. Several studies have focused on various fundamental cellular processes of PLAGL2 and its crucial mechanism in tumorigenesis, but the exact role of PLAGL2 and underlying mechanism in CRC yet remain largely unknown.

Our study revealed that enhanced PLAGL2 expression in CRC tissues is positively correlated with the expression of mesenchymal markers but is inversely correlated with the epithelial marker expression. The data in this study also demonstrated the crucial effects of PLAGL2 on the proliferation, migration and invasion of CRC cells both in vitro and in vivo. Moreover, these results demonstrated that PLAGL2 triggers EMT, contributing to CRC metastasis via β-catenin -dependent regulation of ZEB1. Our findings illustrate that PLAGL2 serves as a crucial regulatory factor of the β-catenin-ZEB1 molecular mechanisms, and may be a promising therapeutic target for CRC anti-metastatic strategies

## Methods

### Patients and specimens

Forty-two pairs of CRC specimens and matched para-carcinoma samples, were randomly selected from patients who had not received chemotherapy or radiotherapy before excision. All samples were gathered from patients who underwent surgery at the Union Hospital (Wuhan, China). The diagnosis of CRC in each case was confirmed by the original histopathological report. Our study protocol (S-082/2019) was approved by the Ethics Committee of Tongji Medical College, Huazhong University of Science and Technology (Wuhan, China).

### Cell culture and reagents

The CRC cell lines (SW620, SW480, LOVO, DLD1 and HCT116) and the normal colon epithelial cell line FHC were purchased from American Type Culture Collection (ATCC, Manassas, VA, USA) and were checked and authenticated for genotypes by DNA fingerprinting within 6 months. The cell lines were incubated in a humidified atmosphere with 5% CO_2_ at 37 °C and cultivated in the recommended growth medium, supplemented with 10% foetal bovine serum (FBS), 100 mg/ml streptomycin and 100 U/mL penicillin (Sigma-Aldrich, St Louis, MO, USA). The GSK-3β inhibitor CHIR-98014, the Akt inhibitor MK2206 and the small-molecule inhibitor PNU75654 were purchased from Selleck (Houston, TX, USA). The Akt activator SC-79 was purchased from MedChem Express (MCE, Monmouth Junction, NJ, USA).

### Western blotting (WB) and co-immunoprecipitation (co-IP) analysis

The WB analysis was performed as previously described.^20^ Antibodies for the WB analysis are shown in Supplementary Table 1. For co-IP assays, whole cell lysates were incubated with primary antibodies at 4 °C for 2 h, and with ProteinA/G PLUS-Agarose beads (Cell Signaling Technology (CST), Danvers, MA, USA) at 4 °C overnight. The agarose beads were gathered, washed with cold phosphate-buffered saline and further detected by the WB analysis.

### Quantitative real-time polymerase chain reaction (qRT-PCR)

Total RNA from CRC cells and specimens was extracted with RNAiso Plus (TaKaRa, Kyoto, Japan). The SYBR® Premix Ex Taq (TaKaRa) was utilised for the qRT-PCR assay. The primers were listed in Supplementary Table 2. The mRNA expression was quantitated using the 2-(△Ct sample–△Ct control) method.

### Cell proliferation assay

2 × 10^3^ cells were seeded into 96-well plates and observed for 120 h. In each sample, the medium with 10% CCK-8 reagents (Dojondo Laboratories, Kumamoto, Japan) was used to replace the original medium at the scheduled time points (24, 48, 72, 96, and 120 h). After incubation at 37 °C for 2 h, the absorbance of each sample was then detected at 450 nm. An EdU cell proliferation assay kit (RiboBio, Guangzhou, China) was also used to further assess the cell growth. 1 × 10^5^ cells were planted in 96-well plates. Briefly, the cells were incubated with 50 µM EdU at 37 °C for 2 h before fixation, permeabilisation and EdU staining. Hoechst 33342 was utilised to counterstain the nuclei at room temperature for 30 min. Cell proliferation was investigated by counting the cells with incorporated EdU and there were 5 samples per group.

### Colony formation assay

Transfected SW480 and LOVO cells were planted in 6-well plates (500 cells/well) and cultured in the recommended growth medium for 2 weeks. Changing culture medium was performed every 3–4 days. We fixed the cell colonies with 4% paraformaldehyde for 15 min, stained the colonies with 1% crystal violet, and then counted the colonies.

### Cell cycle analysis

The transfected cells were harvested for cell cycle analysis, washed with cold PBS, and fixed with 75% cold ethanol. Before analysis with a BD FACS Flow Cytometer, the cells were incubated with propidium iodide (PI) (50 μg/mL, AntGent, Wuhan, China) for 30 min.

### Wound-healing assay

The transfected cells were cultured in 6-well plates. After the cells reached 90% confluence, a standard 200 μl pipette tip was subsequently utilised to scratch linear wounds. In addition, the cell monolayers were cultivated in FBS-free medium. After scratching, the images of the wound closure were captured at 0, 24 and 48 h.

### Transwell migration and invasion assay

8 × 10^4^ cells, suspended in medium without FBS, were seeded into transwell chambers (Costar Corning, Kennebunk, ME, USA), with or without Matrigel (Sigma-Aldrich) coating. The lower chamber contained medium with 10% FBS as chemokine. Twenty-four hours later, the migratory or invasive cells on the lower surface of the chamber were photographed and counted in 10 random microscopic fields after crystal violet staining.

### Immunofluorescence (IF) assay

The IF assay was carried out as described previously.^21^ Primary antibodies specific for E-cadherin (1:100), Vimentin (1:100), N-cadherin (1:100) and ZEB1 (1:100) were obtained from Proteintech (Rosemont, IL, USA). The primary antibody specific for β-catenin (1:150) was obtained from CST (Danvers, MA, USA). The Fluorescence images were captured (Olympus, Tokyo, Japan).

### Immunohistochemistry (IHC)

IHC analysis was conducted as described elsewhere.^22^ The IHC staining results were evaluated by two independent pathologists (double-blinded). Briefly, the percentage of stained tumour cells (0, 0–5%; 1, 6–25%; 2, 26–50%; 3, 50%–100%) and staining intensity scores (0, negative; 1, weak; 2, moderate; 3, strong) were summed. The CRC tissues were categorised into four groups: negative, ≤5% cells stained, regardless of intensity; weak expression, 1–2 points; moderate expression, 3–4 points; and strong expression, 5–6 points. The total score ≥3 was classified as significant overexpression and was considered as positive expression. Antibodies for the IHC analysis are shown in Supplementary Table 1.

### Transfection

Lentiviral vectors with PLAGL2 shRNA and negative control shRNA were acquired from Genechem (Shanghai, China) and utilised in our study: PLAGL2 shRNA 5′-GACCCATGATCCTAACAAA-3′. SW480 was transduced with a lentiviral vector with PLAGL2 shRNA. LOVO was then transfected with a lentivirus carrying full-length PLAGL2 or control sequences (OBiO Technology, Shanghai, China). The knockdown or overexpression of PLAGL2 was detected by qRT-PCR and WB analysis. Short interfering RNAs for ZEB1 and β-catenin were purchased from RiboBio. The sequences for the siRNAs were as follows: siZEB1, 5′-CCTAGTCAGCCACCTTTAA-3′, siβ-catenin, 5′-AUUACAAU CCGGUUGUGA ACGUCCC-3′.

### Chromatin immunoprecipitation (ChIP)

The ChIP assay was performed with a Simple ChIP Plus Enzymatic Chromatin IP Kit (CST), with anti-β-catenin (1:50, CST) and anti-TCF4 (1:50, abcom, Shanghai, china) antibodies. The bound DNA fragments were amplified by qRT-PCR, and then the products of qRT-PCR were examined by gel electrophoresis on 2% agarose gels. Input and IgG were used simultaneously to ascertain that the captured signals were derived from specific bonding. The PCR primers for ZEB1 were follows: forward primer (5′-ATGGACCAATAAATAACG-3′), reverse primer (5′-TCTTCAAACCTTTCAA CT-3′).

### Xenograft assay

Lentivirus carrying specific DNA sequences were transduced into SW480 and LOVO cells. Five-week-old BALB/c male nude mice were purchased from Beijing Vital River Laboratory Animal Technology Co., Ltd. (Beijing, China). To assess the proliferation in vivo, 5 × 10^6^ cells, suspended in 150 μl PBS, were implanted subcutaneously into the groin of the mice. There were 7 mice in each group. Tumour size was measured every 4 days using the following formula: V = 0.5 × (length) × (width)^2^. Mice were sacrificed at 28 days after implantation. To assess the tumour metastasis in vivo, 3 × 10^6^ cells were injected into the tail vein of mice, and all mice were sacrificed after 6 weeks. The care and handling of the mice were approved by the Institutional Animal Care and Use Committee of Tongji Medical College, Huazhong University of Science and Technology.

### Statistical analysis

The data analysis were conducted using a Student’s *t*-test for the comparison between groups. The *χ*^2^ test was utilised to evaluate the association between the protein levels and clinical characteristics. The correlations in the gene expression levels were analysed by Spearman’s rank correlation coefficients. Differences were thought to be significant at *p < 0.05, **p < 0.01 and ***p < 0.001. n.s: no significance. The results were analysed with SPSS 19.0 software (SPSS Inc., Chicago, IL, USA). All in vitro experiments were repeated at least three times.

## Results

### PLAGL2 is overexpressed in CRC

**Fig. 1:**
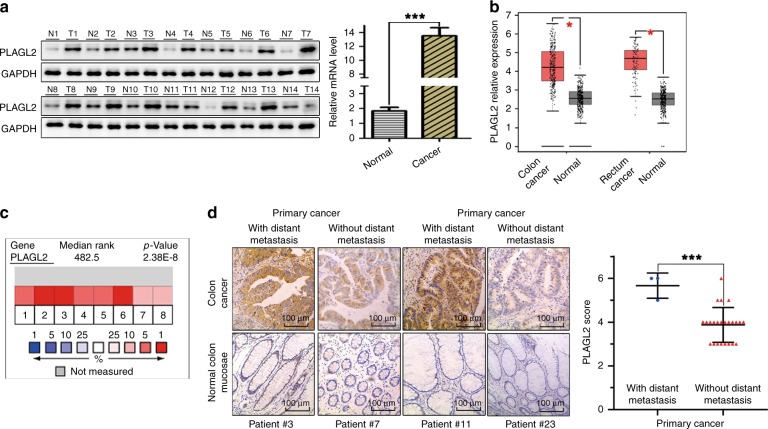
PLAGL2 is overexpressed in CRC. **a** The WB and qRT–PCR analysis showed that PLAGL2 overexpressed in CRC tissues compared to the expression in paired normal samples. T, CRC tissue; N, paired normal tissues. The data are presented as the mean ± SD from three independent experiments. *P < 0.05, **P < 0.01, ***P < 0.001, based on Student’s *t*-test. **b** The box plots were obtained from the GEPIA database to compare the expression of PLAGL2 in CRC specimens and matched normal specimens. **c** Meta-analysis of the PLAGL2 gene expression derived from the Oncomine database. **d** Representative immunohistochemistry images of the PLAGL2 expression levels in primary CRC tumours without metastasis vs CRC tumours with metastasis. Scale bars, 100 μm.

Forty-two CRC specimens and matched adjacent normal colon mucosa were utilised to investigate the aberrant expression of PLAGL2. PLAGL2 was significantly overexpressed in CRC specimens relative to that in matched tissues (Fig. 1a and Supplementary Fig. 1A), which is consistent with the results derived from the Oncomine cancer microarray database (https://www.oncomine.org/resource/main.html)^23^ and the GEPIA database (http://gepia.cancer-pku.cn/detail.php)^24^ (Fig. 1b, c). Interestingly, the CRC samples with distant metastasis exhibited higher PLAGL2 expression than those without distant metastasis (Fig. 1d). In addition, the positive expression rate of EMT-related proteins and their correlation with PLAGL2 in 42 CRC tissues were explored. The data revealed that the expression levels of EMT-related proteins were significantly different between CRC specimens and matched tissues (Supplementary Fig. 1B–D and Table 1). Associations between the expression levels of all of the above-mentioned proteins and clinicopathologic characteristics are shown in Table 2. Notably, the expression of all of these proteins was significantly correlated with the tumour invasion depth and lymph node metastasis. Moreover, PLAGL2 was positively associated with the expression of N-cadherin, Vimentin and β-catenin, but was inversely associated with E-cadherin (Supplementary Fig. 1B–D and Table 3). Collectively, these data suggested that the enhanced PLAGL2 expression in CRC patients was associated with aggressive behaviour.

**Table 1:** Expression of PLAGL2, E-cadherin, N-cadherin, β-catenin and Vimentin in 42 cases of colorectal cancer and adjacent normal mucosa tissues (*X*^2^ test).

Proteins	Colorectal cancer tissues	Adjacent normal mucosa tissues	*P*-value
PLAGL2			
Positive	27	5	0.000
Negative	15	37	
E-cadherin			
Positive	14	26	0.009
Negative	28	16	
N-cadherin			
Positive	25	12	0.004
Negative	17	30	
β-catenin			
Positive	24	14	0.028
Negative	18	28	
Vimentin			
Positive	28	13	0.001
Negative	14	29

**Table 2:** Correlation between PLAGL2, E-cadherin, N-cadherin, vimentin β-catenin expression and clinicopathologic features in 42 cases of CRC tissues (*χ*^2^ test).

Parameters	*n*	PLAGL2			E-cadherin			N-cadherin			Vimentin			β-catenin		
		+	−	*P*	+	−	*P*	+	−	*P*	+	−	*P*	+	−	*P*
Age (years)																
< 60	24	16	8	0.71	9	15	0.51	12	12	0.15	18	6	0.19	16	8	0.15
≥ 60	18	11	7		5	13		13	5		10	8		8	10	
Gender																
Male	26	15	11	0.26	10	16	0.37	15	11	0.76	18	8	0.65	14	12	0.58
Female	16	12	4		4	12		10	6		10	6		10	6	
Size of tumour																
<5 cm	15	9	6	0.67	7	8	0.17	11	4	0.17	12	3	0.17	10	5	0.35
≥5 cm	27	18	9		7	20		14	13		16	11		14	13	
Differentiation																
Well-moderate	19	14	5	0.25	8	11	0.27	10	9	0.41	14	5	0.38	13	6	0.18
Poor	23	13	10		6	17		15	8		14	9		11	12	
T Stages																
T1–T2	15	6	9	0.01	9	6	0.01	5	10	0.01	7	8	0.04	4	11	0.00
T3–T4	27	21	6		5	22		20	7		21	6		20	7	
*Metastasis*																
N Stages																
N0	14	6	8	0.04	8	6	0.02	5	9	0.03	4	10	0.00	5	9	0.047
N1–2	28	21	7		6	22		20	8		24	4		19	9	
M Stages																
M0	38	24	14	0.64	12	26	0.46	22	16	0.51	24	14	0.12	22	16	0.76
M1	4	3	1		2	2		3	1		4	0		2	2

**Table 3:** The correlation between expression levels of PLAGL2 and E-cadherin, N-cadherin, Vimentin and β-catenin in 42 cases of colon cancer tissues by immunohistochemistry (Spearman’s rank correlation).

	PLAGL2			
	+	−	*r*	*P*-value
E-cadherin				
+	6	8	−0.316	0.041
** −**	21	7		
N-cadherin				
+	20	5	0.398	0.009
** −**	7	10		
Vimentin				
** +**	22	6	0.422	0.005
** −**	5	9		
β-catenin				
+	19	5	0.359	0.020
** −**	8	10		

### PLAGL2 promotes the proliferation, migration and invasion of CRC cells in vitro

**Fig. 2:**
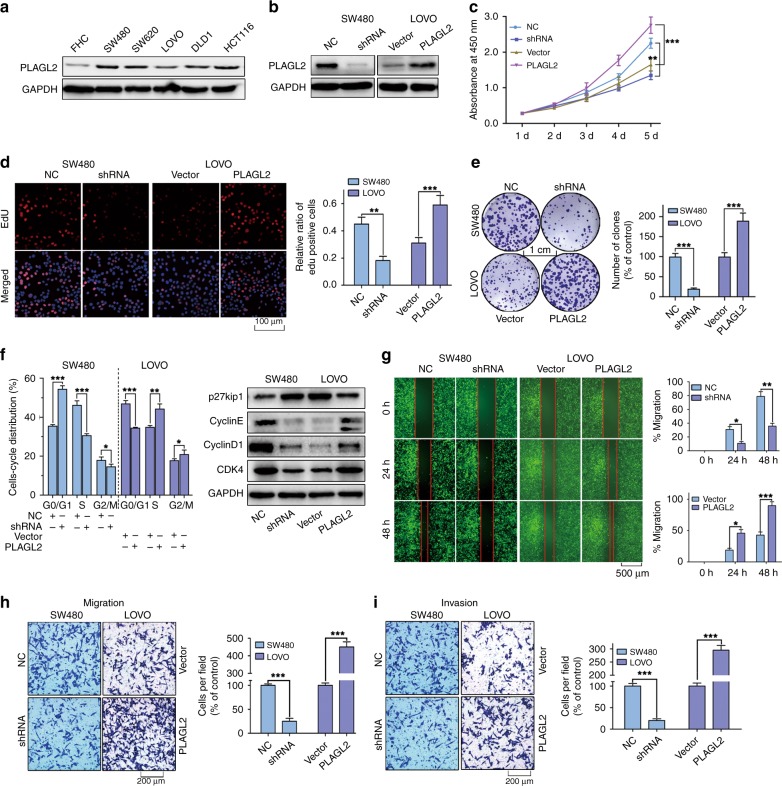
PLAGL2 promotes the proliferation, migration and invasion of CRC cells in vitro. **a** PLAGL2 expression levels in five CRC cell lines and the normal colon epithelial cell line FHC. **b** The effects of PLAGL2 depletion and overexpression were determined by the WB analysis. **c**–**e** The cell proliferation was regulated by modified PLAGL2 expression and was examined by CCK8 (**c**), EdU (**d**) and colony formation assays (**e**) in SW480 and LOVO cells. Scale bars, 100 μm (**d**). Scale bars, 1 cm (**e**). **f** The cell cycle results showed that PLAGL2 regulates cell cycle progression. PLAGL2 depletion increased the G0G1 fraction and decreased the S and G2M fraction. The expression of key cell cycle regulatory proteins was regulated by modified PLAGL2 expression. **g** The migration capacity of PLAGL2 in SW480 and LOVO cells was detected with a wound-healing assay. The cells migrating into the wounded areas were photographed at 0, 24, and 48 h. Scale bars, 500 μm. **h**, **i** The migration and invasion capacity of PLAGL2 in SW480 and LOVO cells were also evaluated with transwell assays. Scale bars, 200 μm. *P < 0.05, **P < 0.01, ***P < 0.001, based on Student’s *t*-test. The data are presented as the mean ± SD from three independent experiments.

All five CRC cell lines exhibited higher PLAGL2 expression than that in the normal colon epithelial cell line FHC (Fig. 2a and Supplementary Fig. 2A). To examine the biological function of PLAGL2, stable PLAGL2-knockdown (SW480) and PLAGL2-overexpression (LOVO) cell lines were established (Fig. 2b and Supplementary Fig. 2B). Both CCK-8 and EdU assays showed that the depletion of PLAGL2 strongly diminished the CRC cell growth compared to that of the controls. By contrast, enhanced PLAGL2 expression significantly promoted the proliferation of LOVO cells (Fig. 2c, d). In addition, the colony formation assay revealed that PLAGL2 overexpression significantly increased the colony numbers relative to those in the controls (Fig. 2e). The elevated expression of PLAGL2 increased the expression of crucial cell cycle proteins, while it diminished p27kip1 expression (a vital cell cycle inhibitor) (Fig. 2f and Supplementary Fig. 2C). Furthermore, the results of cell cycle analysis demonstrated that PLAGL2 depletion increased the G0G1 fraction and decreased the S and G2M fraction. Conversely, PLAGL2 overexpression decreased the G0G1 fraction, and increased the S and G2M fraction (Fig. 2f and Supplementary Fig. 2D–G). In all, these results demonstrate that PLAGL2 promotes the growth of CRC cells in vitro. Meanwhile, the effects of PLAGL2 on the metastatic ability of CRC cells was evaluated with wound-healing and transwell migration assays. These results demonstrated an enhanced invasion and migration ability in cells with enhanced PLAGL2 expression compared to those in the controls (Fig. 2g–i). These results indicate that enhanced PLAGL2 expression contributes to CRC development.

### PLAGL2 promotes CRC cell growth and metastasis in vivo

**Fig. 3:**
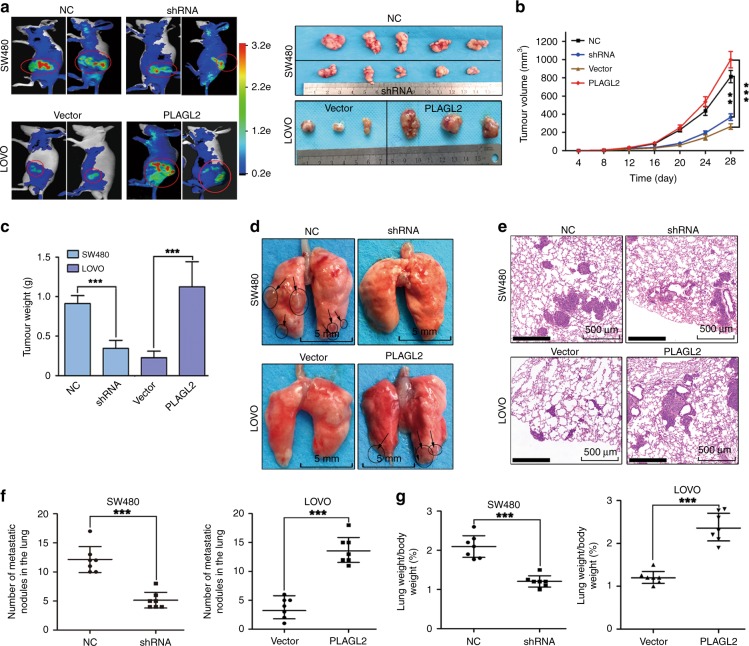
PLAGL2 promotes CRC cell proliferation and metastasis in vivo. **a** Representative bioluminescence pictures of the nude mice 28 days after injection. In all, 5 × 10^6^ stable SW480 and LOVO cells were injected subcutaneously into the groin of nude mice (*n* = 7 per group). Representative images of the corresponding xenograft 28 days after inoculation. **b** Tumour volumes in the different groups. The growth curve of the tumours that formed after subcutaneous injection. **c** Enhanced PLAGL2 expression increased the tumour weights. **d** Lung metastasis models. Representative images of visible lung metastases. The metastatic nodules are indicated with arrows. Scale bars, 5 mm. **e** Representative images of the corresponding HE staining. Scale bars, 500 μm. **f** The numbers of metastatic nodules. **g** The depletion of PLAGL2 significantly reduced the lung weight. *P < 0.05, **P < 0.01, ***P < 0.001, based on Student’s *t*-test.

To evaluate the in vivo effects of PLAGL2 on CRC cell growth, the transfected cells were implanted subcutaneously into the groin of nude mice. The elevated PLAGL2 expression markedly promoted tumour growth in vivo compared to that of the controls (Fig. 3a–c). The experimental lung metastasis assay was utilised to assess the in vivo role of modified PLAGL2 expression in tumour metastasis. Lower PLAGL2 expression strongly reduced the number of metastatic nodules and the lung weight (Fig. 3d–g). Collectively, these results were consistent with the in vitro findings, suggesting that PLAGL2 enforces CRC metastasis in vivo.

### PLAGL2 induces the ZEB1- mediated EMT process and tumorigenesis of CRC

**Fig. 4:**
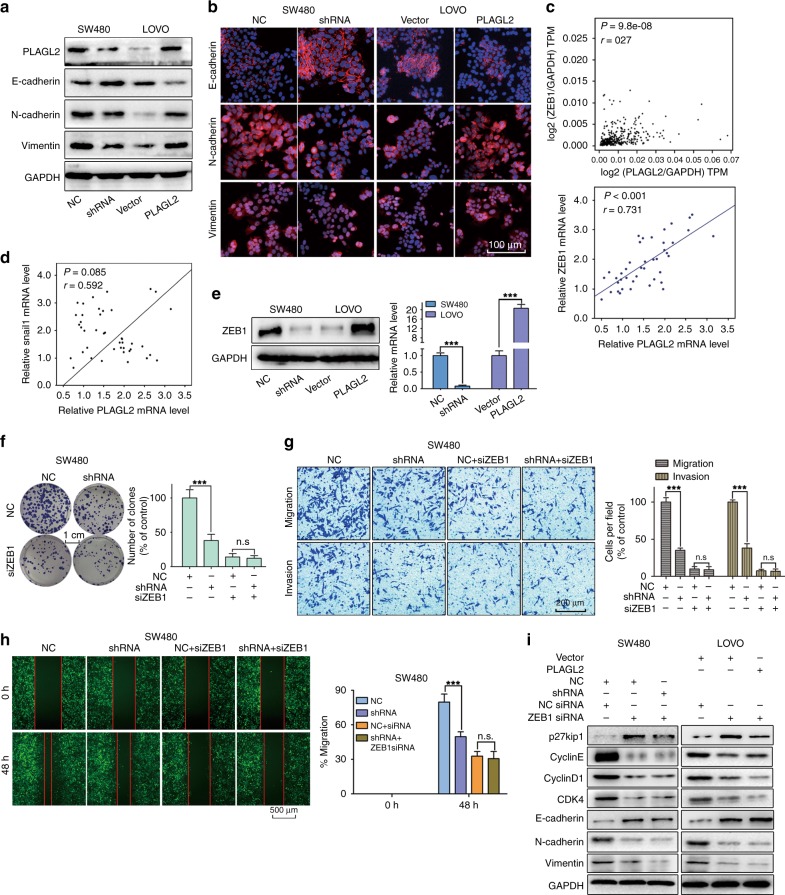
PLAGL2 induces ZEB1-mediated EMT. **a** The levels of three EMT-related proteins in SW480 and LOVO cells. **b** Immunofluorescence assay was performed to detect EMT markers in SW480 and LOVO cells. Targeted proteins were stained red, and the nuclei were stained blue with 4′,6-diamidino-2-phenylindole (DAPI). Scale bars, 100 μm. **c** Data derived from the GEPIA database showed that there was a significant positive correlation between PLAGL2 and ZEB1 in CRC tissues. The data from this study also revealed a significant positive correlation between PLAGL2 and ZEB1. **d** The correlation between PLAGL2 and snail1 was also detected. There was no significant correlation between PLAGL2 and snail1 in CRC tissues. **e** The WB and qRT–PCR analysis showed that enhanced PLAGL2 expression increased ZEB1 expression. **f**–**h** The depletion of PLAGL2 did not further decrease proliferation (**f**), migration (**g**, **h**) and invasion (**g**) in ZEB1-knockdown SW480 cells. **i** The downregulation of ZEB1 could rescue the levels of the EMT-related and cell cycle regulatory proteins in SW480 and LOVO cells, which was examined by the WB analysis. The data are presented as the mean ± SD from three independent experiments. n.s: no significance. *P < 0.05, **P < 0.01, ***P < 0.001, based on Student’s *t*-test.

In consideration of the correlation between enhanced PLAGL2 expression and EMT-related proteins in CRC samples, we wondered whether EMT could account for the PLAGL2-mediated phenotypic changes described above. We demonstrated that PLAGL2 significantly diminished the expression of E-cadherin and elevated the levels of N-cadherin and vimentin compared to those of the controls (Fig. 4a), which was also confirmed by immunofluorescence (Fig. 4b). Furthermore, the involvement of EMT was further investigated by WB and IHC analysis (Supplementary Fig. 3A–B), which were conducted with mouse tumours formed by corresponding cells, indicating that PLAGL2 promotes EMT in vivo. These data suggest that PLAGL2 induces EMT and progression of CRC.

Given the significant inverse association between ZEB1 and E-cadherin across various carcinomas, we also wondered whether ZEB1 was involved in PLAGL2-induced EMT. A significantly positive correlation between PLAGL2 and ZEB1 was observed in CRC tissues (Fig. 4c). Besides, the correlations between PLAGL2 and other EMT transcription factors (snail1, slug, twist1, twist2 and ZEB2), were also explored. As shown in Fig. 4d and Supplemental Fig. 3C. We have not observed a statistically significant correlation between PLAGL2 and any other EMT transcription factor. PLAGL2 remarkably increased the expression of ZEB1 compared to that of the controls (Fig. 4e). Besides, the depletion of PLAGL2 did not further decrease the proliferation, migration and invasion in the ZEB1-knockdown SW480 cells (Fig. 4f–h, Supplementary Fig. 3D–G). PLAGL2 also did not further increase the proliferation, migration and invasion in the ZEB1-knockdown LOVO cells (Supplementary Fig. 3E, Supplementary Figs. 3H and 4A–B). The downregulation of ZEB1 could rescue the levels of the EMT-related and cell cycle regulatory proteins in PLAGL2-depleted SW480 cells and PLAGL2-overexpressed LOVO cells (Fig. 4i and Supplementary Fig. 4C).

Grainyhead-like-2 (GRHL2) is a broad suppressor of oncogenic EMT by inhibiting the TGF-beta signalling pathway and directly inhibiting ZEB1 expression.^25^ ZEB1 repressed GRHL2 expression by directly binding to the GRHL2 promoter.^25–27^ The GRHL2-ZEB1 bidirectional negative feedback loop drives EMT or MET in response to extracellular signals.^26–28^ It was worth exploring Whether GRHL2-ZEB1 reciprocal feedback loop was involved in PLAGL2-mediated EMT process and tumorigenesis of CRC. We demonstrated that GRHL2 was significantly overexpressed in CRC specimens relative to that in matched tissues, which is consistent with the results derived from the GEPIA database (Supplementary Fig. 5A–B). We also detected the correlations between GRHL2 and ZEB1. No statistically significant correlation between GRHL2 and ZEB1 could be observed in our CRC samples, which is consistent with the results derived from the GEPIA database (Supplementary Fig. 5C). Besides, no statistically significant correlation between PLAGL2 and GRHL2 could be observed (Supplementary Fig. 5D). Neither at protein level or at mRNA level, we have not detected any regulatory effect of PLAGL2 on GRHL2 expression (Supplementary Figure 5E). Overall, these data suggest that PLAGL2 plays a role in promoting EMT process and CRC tumorigenesis through ZEB1.

### PLAGL2 regulates β-catenin expression by modulating AKT/GSK-3β signalling

**Fig. 5:**
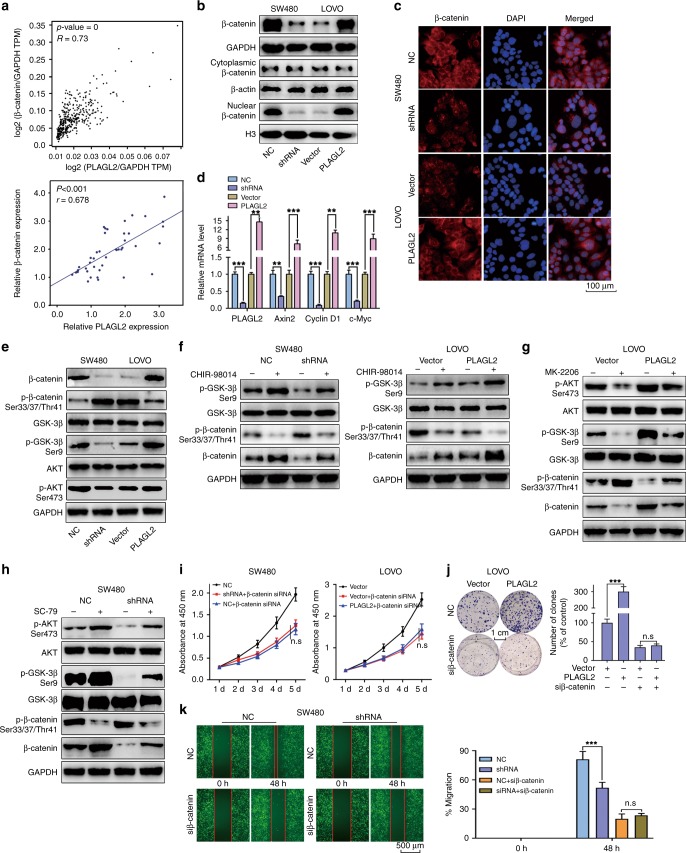
PLAGL2 regulates β-catenin expression by modulating AKT/GSK-3β signalling. **a** The GEPIA database showed that a significantly positive correlation between PLAGL2 and β-catenin could be observed in CRC tissues. The data from this study also revealed a significant positive correlation between PLAGL2 and β-catenin. **b** The nuclear β-catenin and total β-catenin protein levels in SW480 and LOVO cells. GAPDH and H3 were used as cytoplasmic or nuclear protein controls, respectively. **c** The immunofluorescence assays demonstrated that PLAGL2 increased the expression of β-catenin and the nuclear translocation of β-catenin. Scale bars, 100 μm. **d** The depletion of PLAGL2 markedly diminished the expression of the β-catenin target genes Axin2, c-Myc and Cyclin-D1, which were examined by the qRT–PCR analysis. **e** The WB analysis revealed that enhanced PLAGL2 expression promoted AKT and GSK-3β phosphorylation, impeding β-catenin phosphorylation. No significant difference was observed in the total GSK-3β and AKT levels. **f** CHIR98014, a small-molecule GSK-3β inhibitor, partly blocked the effect of modified PLAGL2 expression on β-catenin. **g** Stable LOVO cells were treated with the AKT specific inhibitor MK-2206. The corresponding protein levels were detected by the WB analysis. **h** Stable SW480 cells were treated with the AKT specific activator SC79. The corresponding protein levels were detected by the WB analysis. **i**–**k** The downregulation of β-catenin could rescue the regulatory effect of PLAGL2 on cell proliferation and migration. The cell proliferation was examined by CCK8 (**i**) and colony formation assays (**j**). Scale bars, 1 cm. The migration capacity was detected with a wound-healing assay (**k**). Scale bars, 500 μm. The data are presented as the mean ± SD from three independent experiments. n.s: no significance. *P < 0.05, **P < 0.01, ***P < 0.001, based on Student’s *t*-test.

β-Catenin is a hub molecule of the Wnt/β-catenin signalling pathway, which is involved in EMT and cancer cell metastasis. PLAGL2 was positively correlated with β-catenin expression (Fig. 5a, Tables 2 and 3). Besides, PLAGL2 increased expression of total β-catenin and β-catenin nuclear translocation, which was confirmed by immunofluorescence assays (Fig. 5b, c). The depletion of PLAGL2 markedly diminished the expression levels of the β-catenin target genes Axin2, c-Myc and cyclin-D1 compared to those of the controls (Fig. 5d).

GSK-3-mediated β-catenin phosphorylation and degradation is the main method of regulating β-catenin expression levels. AKT phosphorylation can inactivate GSK-3β. Therefore, the phosphorylation status of β-catenin, AKT and GSK-3β was detected. Compared to the controls, enhanced expression of PLAGL2 promoted AKT and GSK-3β phosphorylation, impeding β-catenin phosphorylation. No significant difference was observed in the total AKT and GSK-3β levels in response to enhanced PLAGL2 expression compared to those in the controls (Fig. 5e). To further determine whether PLAGL2 exerts its function through GSK-3β, a small-molecule GSK-3β inhibitor (CHIR98014) was utilised. CHIR98014 partly blocked the effect of modified PLAGL2 expression on β-catenin (Fig. 5f). Moreover, the AKT specific inhibitor MK-2206 and the activator SC79 were also used to reveal that AKT/GSK-3β is essential for the PLAGL2-induced regulation of β-catenin levels (Fig. 5g, h).

Besides, the depletion of β-catenin could diminish the effect of PLAGL2 on CRC cells proliferation, migration and invasion (Fig. 5i–k and Supplementary Fig. 6A–C). The downregulation of β-catenin also rescued the levels of the EMT-related and cell cycle regulatory proteins in PLAGL2-depleted SW480 cells and PLAGL2-overexpressed LOVO cells (Supplementary Fig. 6D). Taken together, PLAGL2 induces β-catenin expression by modulating AKT/GSK-3β signalling.

### PLAGL2 modulates ZEB1 expression through a β-catenin-dependent pathway

PLAGL2 induces the expression of ZEB1 and β-catenin. The β-catenin-TCF complex modulates transcriptionally ZEB1 expression. These results prompted us to ascertain whether PLAGL2 modulates ZEB1 expression through a β-catenin- dependent pathway.

**Fig. 6:**
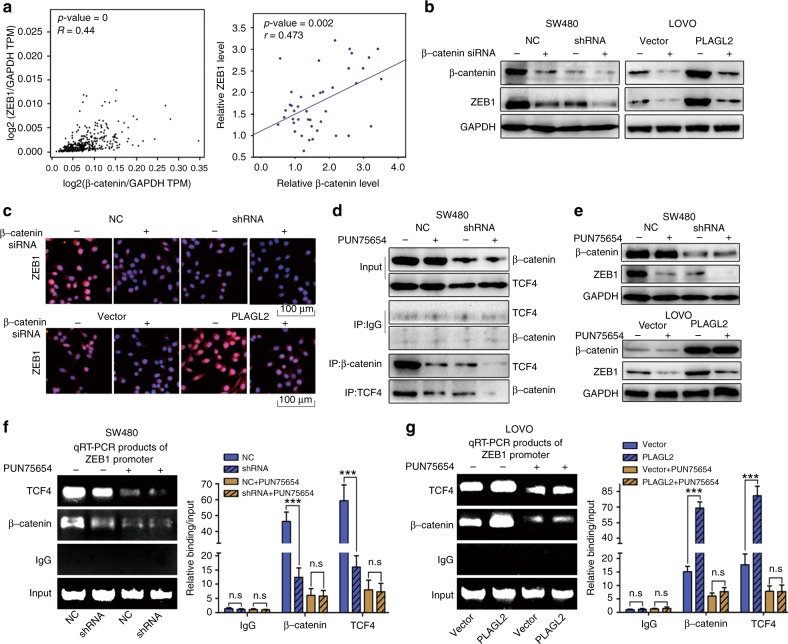
PLAGL2 modulates ZEB1 expression through a β-catenin-dependent pathway. **a** The GEPIA database showed that a significant positive correlation between ZEB1 and β-catenin could be observed in CRC tissues. The data from this study also revealed a significant positive correlation between ZEB1 and β-catenin. **b** The WB analysis showed that the depletion of β-catenin neutralised the promotion of ZEB1 caused by enhanced PLAGL2 expression. (**c**) Depletion of β-catenin neutralised the promotion of ZEB1 caused by enhanced PLAGL2 expression, which was confirmed by immunofluorescence assays. Scale bars, 100 μm. **d** The validity of the small-molecule inhibitor PNU-74654 was verified by the WB analysis. **e** The WB analysis revealed that the PNU-74654, which blocked the interaction between β-catenin and TCF4, thereby neutralising the promotion of ZEB1 caused by enhanced PLAGL2 expression. **f**, **g** The ChIP assays were performed to verify the binding between β-catenin/TCF4 complexes and ZEB1 promoter in SW480 and LOVO cells. The bound DNA fragments were amplified by qRT-PCR. Then the products of qRT-PCR were examined by gel electrophoresis on 2% agarose gels. PNU-74654 blocked β-catenin/TCF4 complexes from directly binding to the ZEB1 promoter and neutralised the promotion of ZEB1 caused by enhanced PLAGL2 expression. The data are presented as the mean ± SD from three independent experiments. n.s: no significance. *P < 0.05, **P < 0.01, ***P < 0.001, based on Student’s *t*-test.

To prove this hypothesis, we examined the correlation between β-catenin and ZEB1 expression. β-Catenin was also positively correlated with ZEB1 expression (Fig. 6a). In addition, compared to the controls, the depletion of β-catenin reversed the promotion of ZEB1 that was caused by enhanced PLAGL2 expression, and further decreased the ZEB1 levels in the cells with lower PLAGL2 expression (Fig. 6b, c). The small-molecule inhibitor PNU-74654, which block the interaction between β-catenin and TCF4, also reversed the regulation of ZEB1 resulting from modified PLAGL2 expression (Fig. 6d, e). These results demonstrated that PLAGL2 modulates ZEB1 expression through a β-catenin- dependent pathway. Specifically, PNU-74654 blocked β-catenin/TCF4 complexes from directly binding to the ZEB1 promoter (Fig. 6f, g), which impaired regulation of PLAGL2 on ZEB1 expression. In all, these results illustrate that PLAGL2 modulates ZEB1 expression through a β-catenin-dependent pathway.

## Discussion

Here we identify the signalling mechanism through which elevated PLAGL2 expression involved in CRC progression. The data in this study illustrate that PLAGL2 induces EMT and an aggressive phenotype in CRC through β-catenin-dependent regulation of ZEB1. Tumour invasion and metastasis, regarded as the most important feature of malignant tumours, are complex and multistep processes.^29,30^ EMT is thought to initiate the early steps of these processes and has traditionally been deemed to a binary process involving a complete conversion from epithelial to mesenchymal state. Nevertheless, it has been increasingly accepted that EMT also includes a range of hybrid states, a phenotype known as “partial EMT’’ (P-EMT).^31–33^ Because p-EMT is not well defined at the molecular level,^34^ it is not clear whether this hybrid status represents an intermediate phase in the process of a mesenchymal transition or its own terminal state.^31^ Therefore, our research mainly focused on the molecular mechanisms of PLAGL2 inducing EMT and an aggressive phenotype in CRC. The EMT subtype has not been thoroughly explored and further research is needed. In this study, we discovered that PLAGL2 was remarkably overexpressed in CRC samples compared to the expression in control samples, and was correlated with worse clinical outcomes. Enhanced PLAGL2 expression was significantly associated with the expression of EMT-related proteins in CRC. Specifically, PLAGL2 promoted the expression of mesenchymal markers and impeded the expression of an epithelial marker (E-cadherin). These results were confirmed by a xenograft tumour assay, indicating that PLAGL2 modulates EMT in vivo. Previous studies have demonstrated that PLAGL2 impeded differentiation in neural stem cells and gliomas.^12^ PLAGL2 modified the proliferation of haematopoietic progenitor and leukaemia cells,^15^ and promoted cell migration and invasion in various cancers.^16,35^ The data in our present study showed that PLAGL2 promoted the proliferative and metastatic properties of CRC cells in vitro. Similarly, the xenograft assay also indicated that PLAGL2 enforced the metastasis and tumorigenesis of CRC.

Recent studies have described ZEB1 as a vital activator of the EMT process that mediates the EMT-related protein expression.^36,37^ ZEB1 also induced tumorigenesis by impeding the expression of stemness-inhibiting microRNAs.^38^ In addition, ZEB1 was involved in 5-fluorouracil resistance in CRC cells.^39^ Given that ZEB1 plays a crucial role in CRC progression, it is urgent to reveal the potential signalling pathway that regulate the expression of ZEB1. Many oncogenes have been implicated in the regulation of ZEB1 expression. Myocyte enhancer factor 2D(MEF2D) transduced multiple signals that activated ZEB1 expression and EMT, promoting metastasis in CRC.^40^ Polypyrimidine tract binding protein 3(PTBP3) promoted EMT in breast cancer by regulating the ZEB1 mRNA stability.^41^ GRHL2 protein was the first direct transcriptional repressor of the ZEB1 gene to be reported.^25^ In different biological backgrounds, the function of GRHL2 is not consistent, sometimes even completely opposite. The GRHL2/ZEB1 feedback loop has been reported to control EMT/MET primarily in breast cancer,^26–28^ but no statistically significant correlation between GRHL2 and ZEB1 could be observed in the present study. In this study, a positive correlation between PLAGL2 and ZEB1 was observed in CRC samples, but we have not seen a statistically significant correlation between PLAGL2 and any other EMT transcription factor. We demonstrated that the expression of ZEB1 was diminished by the depletion of PLAGL2. The data also illustrated that ZEB1 is required for PLAGL2 to function as an oncogene in CRC. These results indicate that ZEB1 is a vital target gene of PLAGL2.

The β-catenin signalling pathway plays a significant role in the EMT process and is activated by the overexpression or by β-catenin nuclear translocation. The dysregulation of this signalling pathway involves in numerous biological processes, including cell apoptosis,^42^ cell migration^43^ and cell autophagy.^44^ Mutations in crucial regulatory factors of the β-catenin signal mechanism have been widely noted in CRC. The nuclear translocation of β-catenin impeded the expression of E-cadherin and subsequently activated the EMT process. The effects of EMT-TFs on the induction of EMT could be blocked by the depletion of β-catenin. Microtubule-associated serine/threonine kinase like (MASTL) induced CRC progression and chemoresistance by activating the β-catenin signalling pathway.^45^ The ring finger protein 6(RNF6)-mediated degradation of transducin-like enhancer of split 3 (TLE3) remarkably impeded the binding of TLE3 with TCF4/LEF, which promoted the recruitment of β-catenin to TCF4/LEF and activated β-catenin signalling in CRC.^46^ Our study demonstrated that PLAGL2 was significantly associated with the expression of β-catenin. In addition, the elevated expression of PLAGL2 resulted in the overexpression of β-catenin and in the nuclear translocation of β-catenin. Moreover, PLAGL2 induced AKT phosphorylation, thereby enforcing GSK-3β phosphorylation and inactivating its activity. Due to the inactivation of GSK-3β, β-catenin degradation was diminished, and the cellular β-catenin levels increased. Taken together, PLAGL2 induced β-catenin expression by regulating AKT/GSK-3β signalling. However, further investigation is warranted to identify the molecular mechanisms driving the PLAGL2 regulation of AKT phosphorylation.

Interestingly, ZEB1 was demonstrated to be a vital activator in the EMT process, and the β-catenin pathway was involved in EMT. Previous studies have shown that β-catenin forms a transcriptional activation complex with TCF4, then binding to the ZEB1 promoter region and inducing its expression.^9^ TP53BP2 prevented β-catenin from activating the expression of ZEB1 by forming a TP53BP2-β-catenin- E-cadherin complex, maintaining the plasticity of epithelial cells and suppressing metastasis.^8^ Our study showed that β-catenin was also positively correlated with ZEB1 expression. The depletion of β-catenin neutralised the regulation of ZEB1 expression that was caused by modified PLAGL2 expression. The inhibitor PNU-74654 had almost identical effects as β-catenin depletion on the regulation of the expression of ZEB1.

Overall, this study illustrates that enhanced PLAGL2 expression in CRC is positively associated with overexpressed N-cadherin and Vimentin, and is inversely correlated with the expression of E-cadherin. PLAGL2 increases AKT and GSK-3β phosphorylation. The inactivation of GSK-3β reduces β-catenin degradation and elevates β-catenin levels, promoting β-catenin nuclear translocation. Elevated β-catenin forms a transcriptional activation complex with TCF4, then binding to the promoter region of ZEB1 and inducing its expression. Thus, PLAGL2 induces EMT and an aggressive phenotype in CRC through β-catenin-dependent regulation of ZEB1.

## Supplementary information

the supplementary files

## Data Availability

The datasets generated and/or analysed during the current study are not publicly available but are available from the corresponding author on reasonable request.
