# Supplementary material for: PLAGL2 promotes epithelial–mesenchymal transition and mediates colorectal cancer metastasis via β-catenin-dependent regulation of ZEB1
Source: Br J Cancer. 2019 Dec 12;122(4):578–89. doi: 10.1038/s41416-019-0679-z (PMC7028997; doi:10.1038/s41416-019-0679-z)
Supplement: Supplementary file 1 — the supplementary files [file 41416_2019_679_MOESM1_ESM.docx]

**Supplementary files online**

1. **Supplementary Figure 1.** Relative expression levels of PLAGL2 and EMT markers in CRC specimens and matched tissues. Scale bars, 100μm.
2. **Supplementary Figure 2.** PLAGL2 promotes the proliferation, migration and invasion of CRC cells in vitro.
3. **Supplementary Figure 3.** PLAGL2 induces the ZEB1-mediated EMT process.
4. **Supplementary Figure 4.** PLAGL2 induces the ZEB1-mediated EMT process.
5. **Supplementary Figure 5.** PLAGL2 induces the ZEB1-mediated EMT process.
6. **Supplementary Figure 6.** The downregulation of β-catenin rescues the regulatory effect of PLAGL2 on cell proliferation and migration.
7. **Supplementary** **Tables: Table 1.** Primary antibodies used in this study

**Table2.** Sequence of primers

1. **Supplementary Figure 1.** Relative expression levels of PLAGL2 and EMT markers in CRC specimens and matched tissues.


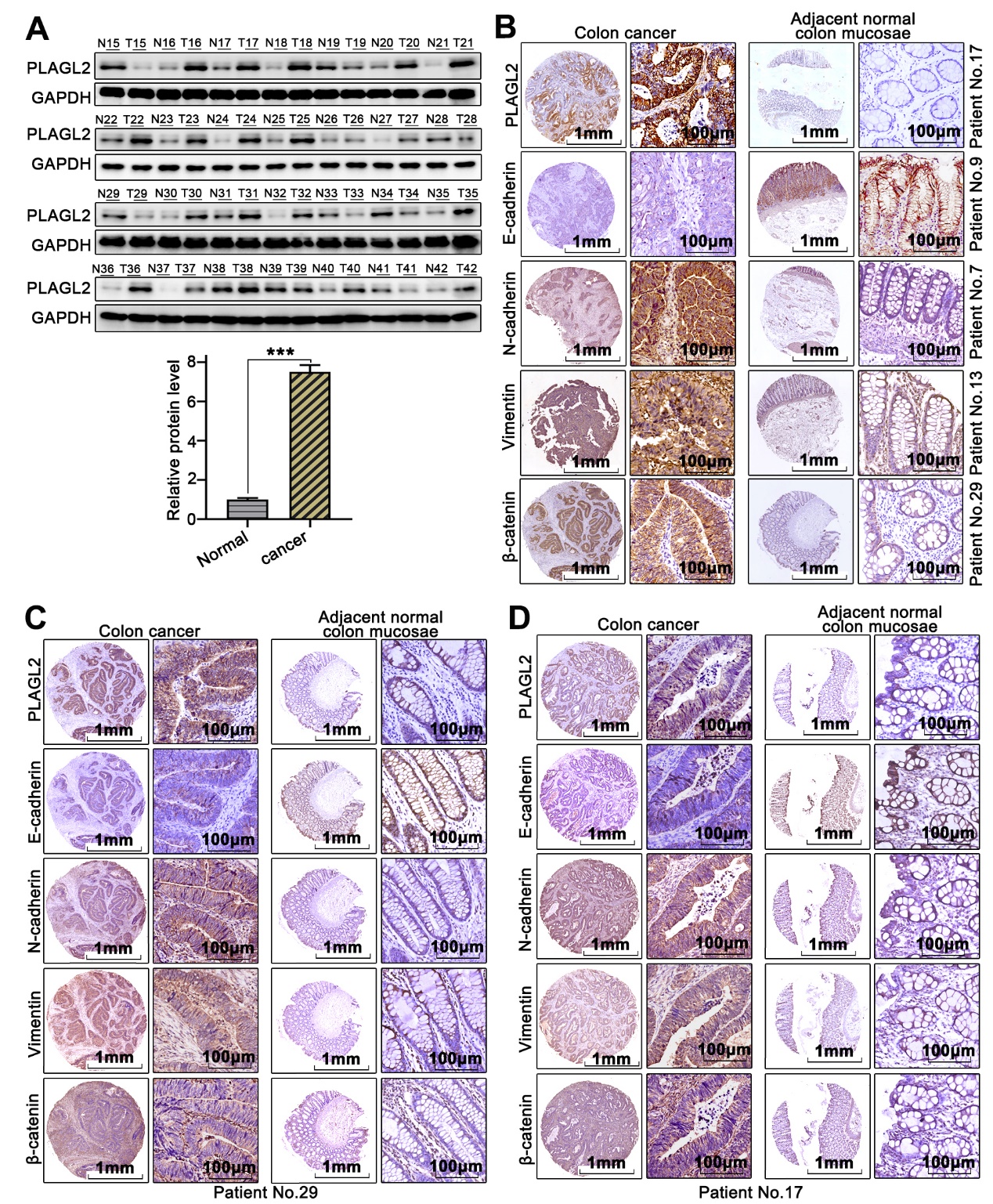


**Supplementary Figure 1.** Relative expression levels of PLAGL2 and EMT markers in CRC specimens and matched tissues. (A) The WB analysis showed that PLAGL2 overexpressed in CRC tissues compared to the expression in paired normal samples. (B) Representative immunohistochemistry images of PLAGL2, E-cadherin, N-cadherin, Vimentin and β-catenin performed with colon cancer tissues and matched normal colon mucosae. Scale bars, 100μm. (C-D) Representative immunohistochemistry images of PLAGL2, E-cadherin, N-cadherin, Vimentin and β-catenin performed with colon cancer tissues and normal colon mucosae of two patients (No.17 and No.29). Scale bars, 100μm.

1. **Supplementary Figure 2.** PLAGL2 promotes the proliferation, migration and invasion of CRC cells in vitro.


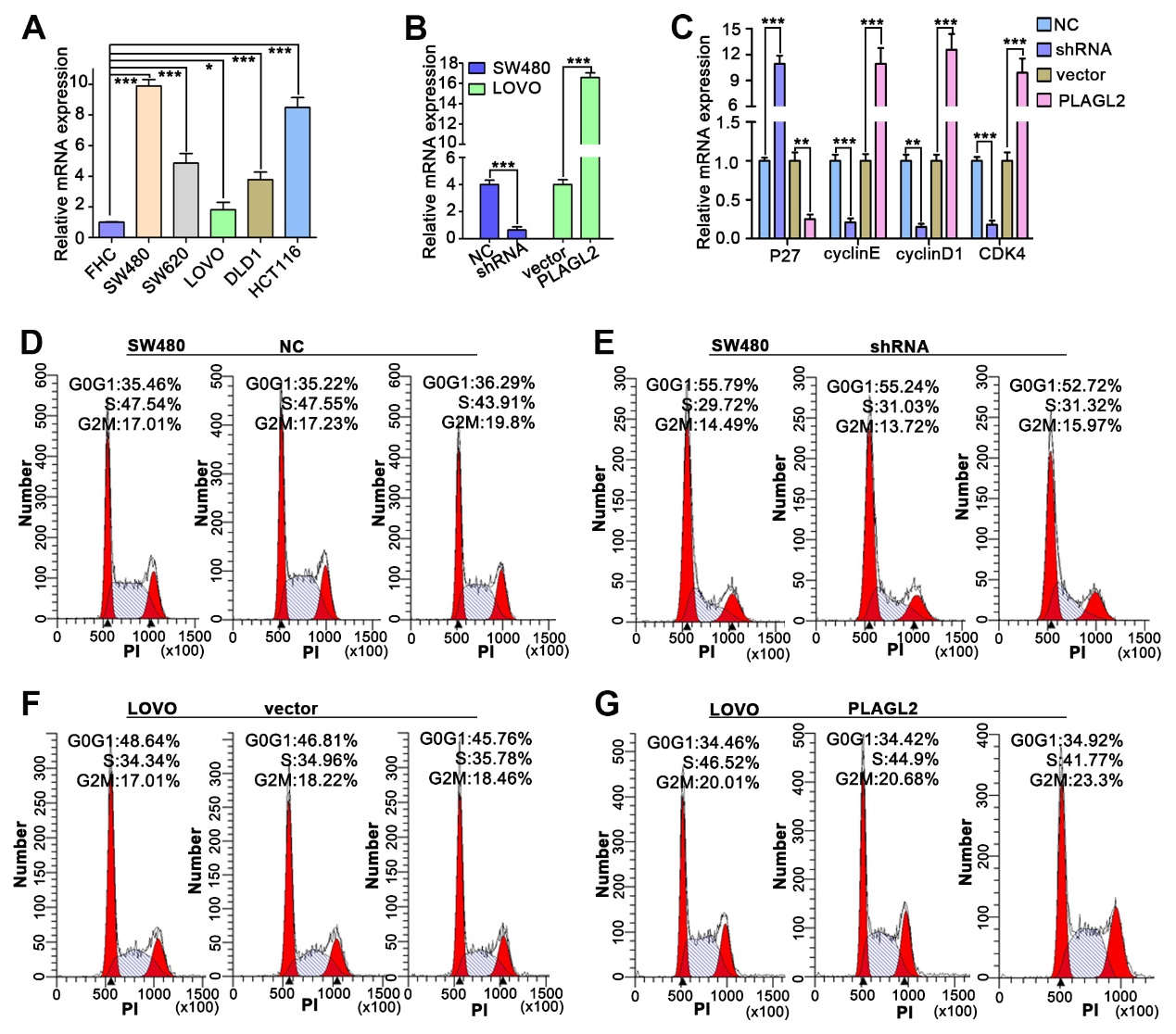


**Supplementary Figure 2.** PLAGL2 promotes the proliferation, migration and invasion of CRC cells in vitro. (A) PLAGL2 mRNA levels in five CRC cell lines and the normal colon epithelial cell line FHC. (B) The effects of PLAGL2 depletion and overexpression were determined by qRT–PCR. (C) Modified PLAGL2 expression affected the mRNA expression of key cell cycle regulatory factors. (D-G) The cell cycle results showed that PLAGL2 regulates cell cycle progression. PLAGL2 depletion increased the G0G1 fraction, and decreased the S and G2M fraction. The data are presented as the mean ± SD from three independent experiments. *P<0.05, **P<0.01, ***P<0.001, based on Student’s t-test.

1. **Supplementary Figure 3.** PLAGL2 induces the ZEB1- mediated EMT process.


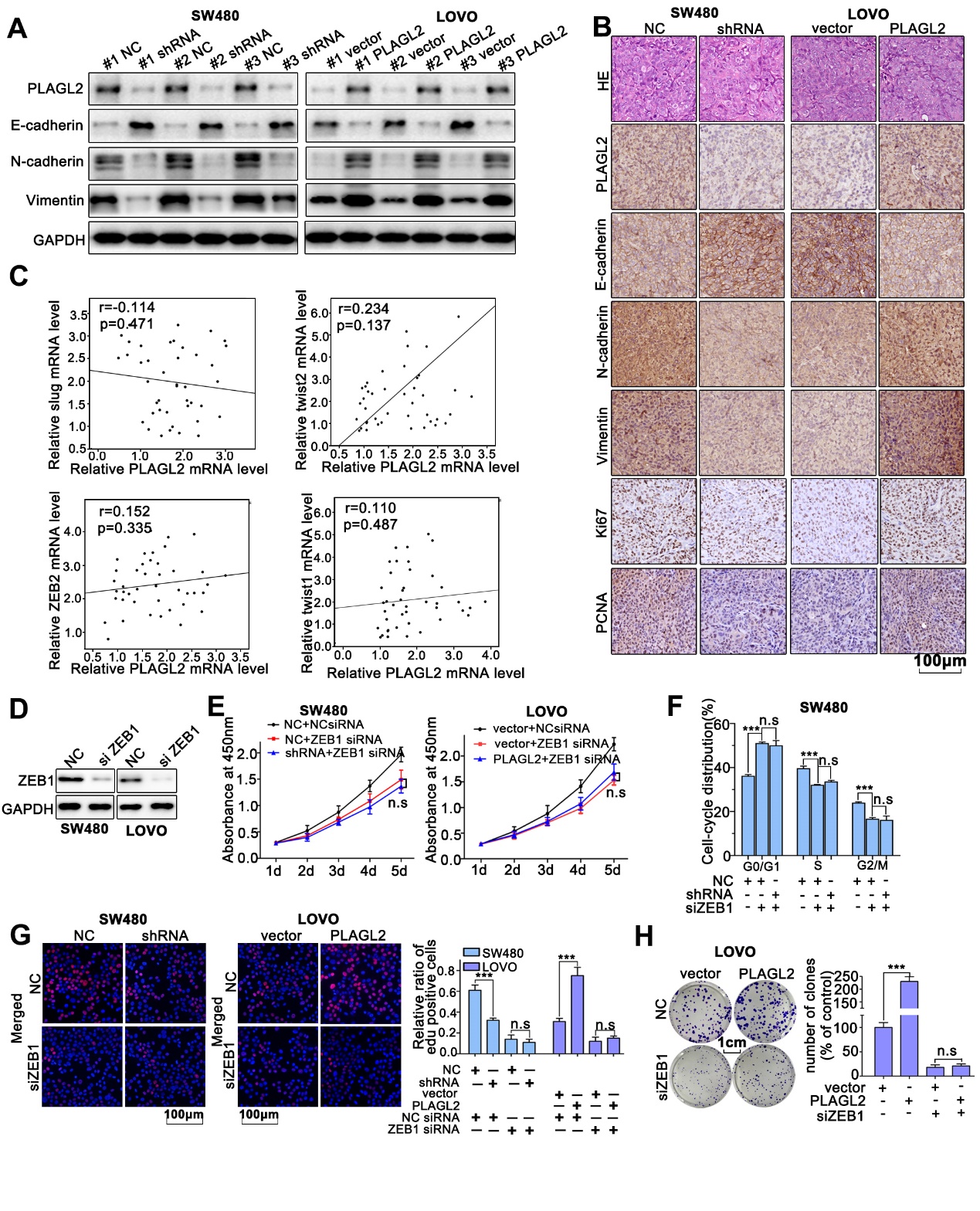


**Supplementary Figure 3.** PLAGL2 induces the ZEB1- mediated EMT process. (A) EMT-related proteins in mouse tumour tissues were detected by the WB analysis. (B) Representative expression of PLAGL2 and three EMT markers and cell proliferation markers in mouse tumour tissues were examined by immunohistochemistry. Scale bars, 100μm. (C) The correlations between PLAGL2 and other EMT transcription factors (slug, twist1, twist2 and ZEB2), were also explored. We have not observed a statistically significant correlation between PLAGL2 and any other EMT transcription factor. (D) The validity of the small interference RNA for ZEB1(siZEB1) was verified by the WB analysis. (E) CCK8 showed that the downregulation of ZEB1 blocked the cell proliferation that was regulated by PLAGL2. (F) The cell cycle results showed that the downregulation of ZEB1 could rescue the regulatory effect of PLAGL2 on cell cycle progression. (G-H) EdU (G) and colony formation (H) assays illustrated that ZEB1 depletion blocked the cell proliferation, regulated by PLAGL2. Scale bars, 100μm(G). Scale bars, 1cm(H). The data are presented as the mean ± SD from three independent experiments. n.s: no significance. *P<0.05, **P<0.01, ***P<0.001, based on Student’s t-test.

1. **Supplementary Figure 4.** PLAGL2 induces the ZEB1-mediated EMT process.


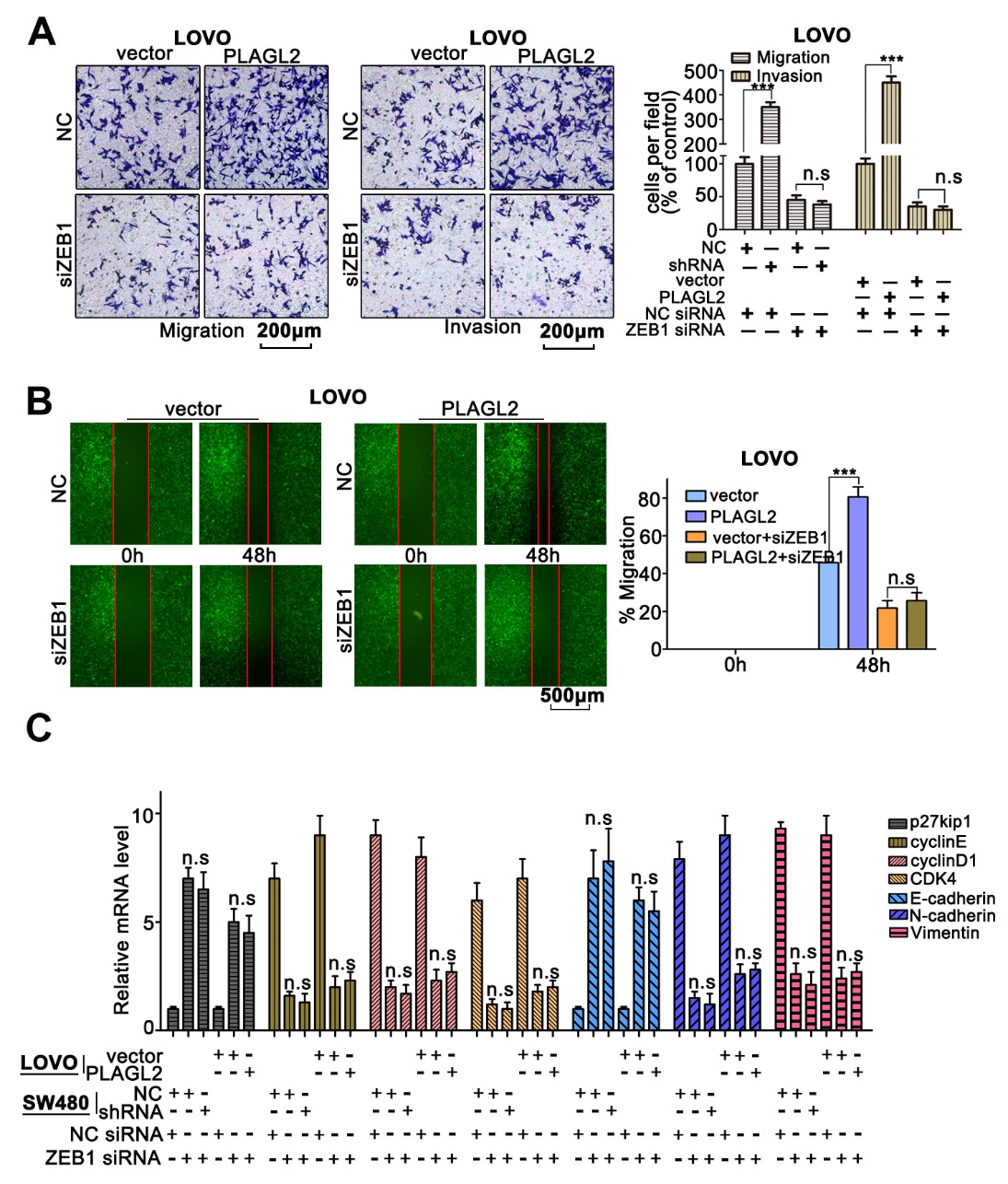


**Supplementary Figure 4.** PLAGL2 induces the ZEB1-mediated EMT process. (A-B) The elevated expression of PLAGL2 did not further increase migration (A) and invasion (B) in ZEB1- knockdown LOVO cells. Scale bars, 200μm(A). Scale bars, 500μm(B). (C) The qRT–PCR analysis showed that the downregulation of ZEB1 could rescue the levels of the EMT-related and cell cycle regulatory proteins in SW480 and LOVO cells. The data are presented as the mean ± SD from three independent experiments. n.s: no significance. *P<0.05, **P<0.01, ***P<0.001, based on Student’s t-test.

1. **Supplementary Figure 5.** PLAGL2 induces the ZEB1-mediated EMT process.


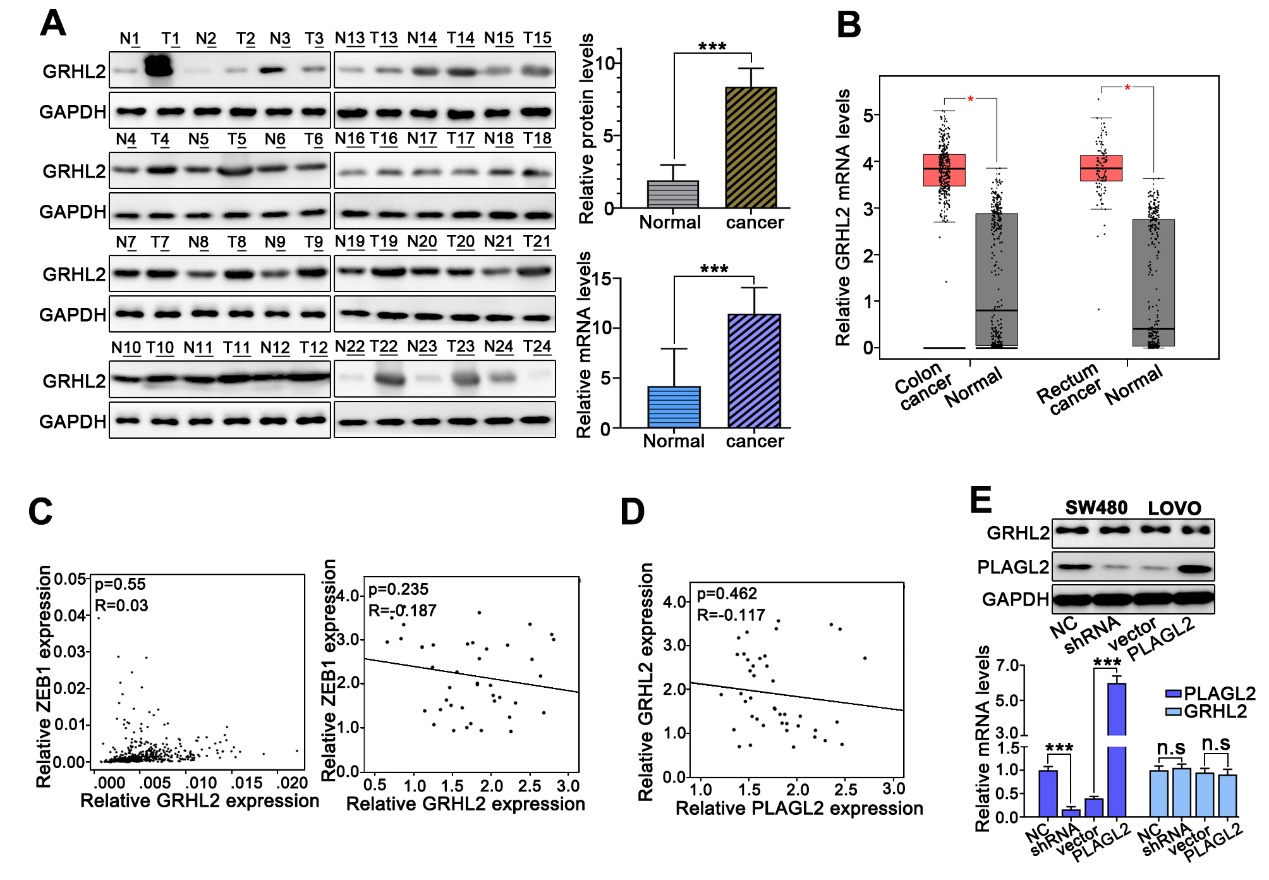


**Supplementary Figure 5.** PLAGL2 induces the ZEB1-mediated EMT process. (A) The WB and qRT–PCR analysis showed that GRHL2 overexpressed in CRC tissues compared to the expression in paired normal samples. (B) The box plots were obtained from the GEPIA database to compare the expression of GRHL2 in CRC specimens and matched normal specimens. (C) The GEPIA database showed that no statistically significant correlation between GRHL2 and ZEB1 could be observed in CRC tissues. The data from this study also revealed no statistically significant correlation between GRHL2 and ZEB1. (D) no statistically significant correlation between PLAGL2 and GRHL2 could be observed. (E) The WB and qRT–PCR analysis were performed to detect the regulatory effect of PLAGL2 on GRHL2 expression. The data are presented as the mean ± SD from three independent experiments. n.s: no significance. *P<0.05, **P<0.01, ***P<0.001, based on Student’s t-test.

1. **Supplementary Figure 6.** The downregulation of β-catenin rescues the regulatory effect of PLAGL2 on cell proliferation and migration.


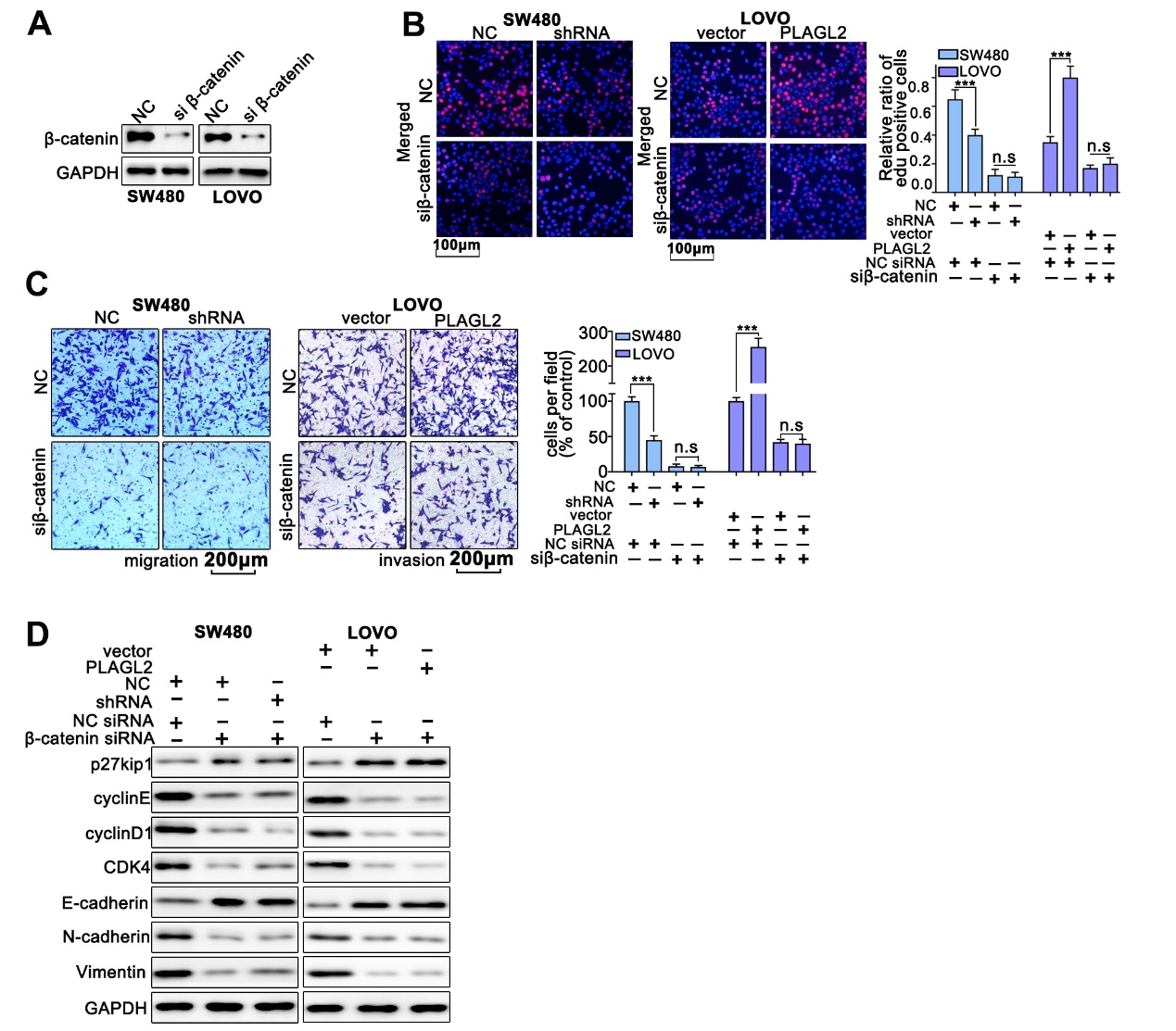


**Supplementary Figure 6.** The downregulation of β-catenin rescues the regulatory effect of PLAGL2 on cell proliferation and migration. (A) The validity of the small interference RNA for β-catenin (siβ-catenin) was verified by the WB analysis. (B) The EdU assays illustrated that β-catenin depletion blocked the cell proliferation, regulated by PLAGL2. Scale bars, 100μm. (C) The β-catenin depletion blocked the cell migration and invasion, regulated by PLAGL2. Scale bars, 200μm. (D) The downregulation of β-catenin could rescue the levels of the EMT-related and cell cycle regulatory proteins in SW480 and LOVO cells, which was examined by the WB analysis. The data are presented as the mean ± SD from three independent experiments. n.s: no significance. *P<0.05, **P<0.01, ***P<0.001, based on Student’s t-test.

1. **Supplementary Tables.**

**Supplementary table1.** Primary antibodies used in this study

| Antibody | Concentration | Specificity | Company |
| --- | --- | --- | --- |
| PLAGL2 | 1:1000(WB); 1:200(IHC) | Rabbit polyclonal | Abcam |
| E-cadherin | 1:1000(WB); 1:200(IHC) | Mouse monoclonal | Proteintech |
| Vimentin | 1:1000(WB); 1:500(IHC) | Mouse monoclonal | Proteintech |
| N-cadherin | 1:1000(WB); 1:300(IHC) | Rabbit monoclonal | Proteintech |
| β-catenin | 1:1000(WB); 1:250(IHC) | Rabbit monoclonal | CST |
| P27kip1 | 1:1000(WB) | Mouse monoclonal | CST |
| Cyclin-E | 1:1000(WB) | Rabbit monoclonal | CST |
| H3 | 1:2000(WB) | Rabbit monoclonal | Proteintech |
| GAPDH | 1:2000(WB) | Rabbit monoclonal | Proteintech |
| ZEB1 | 1:1000(WB); | Rabbit monoclonal | Proteintech |
| TCF4 | 1:1000(WB) | Rabbit monoclonal | Abcam |
| CDK4 | 1:1000(WB) | Rabbit monoclonal | Proteintech |
| Cyclin-D1 | 1:1000(WB) | Mouse monoclonal | Proteintech |
| p-β-catenin | 1:1000(WB) | Rabbit polyclonal | CST |
| p-GSK-3β | 1:500(WB) | Rabbit polyclonal | CST |
| p-AKT | 1:1000(WB) | Rabbit monoclonal | CST |
| GSK-3β | 1:1000(WB) | Rabbit monoclonal | CST |
| Ki67 | 1:8000(IHC) | Rabbit polyclonal | Proteintech |
| PCNA | 1:1000(IHC) | Mouse monoclonal | Proteintech |
| GRHL2 | 1:1000(WB) | Mouse polyclonal | Abcam |
| AKT | 1:1000(WB) | Mouse monoclonal | CST |

**Supplementary table2.** Sequence of primers

| Gene | Forward primer (5’------3’) | Reverse primer (5’------3’) |
| --- | --- | --- |
| PLAGL2 | GAGTCAAGTGAAGTGCCAATGT | TGAGGGCAGCTATATGGTCTC |
| E-cadherin | CGAGAGCTACACGTTCACGG | GGGTGTCGAGGGAAAAATAGG |
| Vimentin | CGAAACTTCTCAGCATCACG | GCAGAAAGGCACTTGAAAGC |
| N-cadherin | TCAGGCGTCTGTAGAGGCTT | ATGCACATCCTTCGATAAGACTG |
| P27kip1 | AACGTGCGAGTGTCTAACGG | CCCTCTAGGGGTTTGTGATTCT |
| Cyclin-D1 | GCTGCGAAGTGGAAACCATC | CCTCCTTCTGCACACATTTGAA |
| Cyclin-E | AAGGAGCGGGACACCATGA | ACGGTCACGTTTGCCTTCC |
| CDK4 | TCAGCACAGTTCGTGAGGTG | GTCCATCAGCCGGACAACAT |
| Axin2 | TACACTCCTTATTGGGCGATCA | TTGGCTACTCGTAAAGTTTTGGT |
| Cyclin-D1 | GTGCTGCGAAGTGGAAACC | ATCCAGGTGGCGACGATCT |
| c-Myc | TTCGGGTAGTGGAAAACCAG | CAGCAGCTCGAATTTCTTCC |
| ZEB1 | CAGCTTGATACCTGTGAATGGG | TATCTGTGGTCGTGTGGGACT |
| Snail1 | TCGGAAGCCTAACTACAGCGA | AGATGAGCATTGGCAGCGAG |
| Twist1 | GTCCGCAGTCTTACGAGGAG | GCTTGAGGGTCTGAATCTTGCT |
| ZEB2 | CAAGAGGCGCAAACAAGCC | GGTTGGCAATACCGTCATCC |
| Slug | CGAACTGGACACACATACAGTG | CTGAGGATCTCTGGTTGTGGT |
| Twist2 | GAGCGACGAGATGGACAATAA | ATGCGCCACACGGAGAA |
| GRHL2 | CCGGCTGCTGCTACTACTAC | GCACTCGGTTTTCTCCTCCA |
| GAPDH | AGAAGGCTGGGGCTCATTTG | AGGGGCCATCCACAGTCTTC |
